# mRNA vaccines against viral pathogens: molecular design, delivery systems, and clinical applications

**DOI:** 10.3389/fmicb.2026.1821852

**Published:** 2026-06-11

**Authors:** Wei Xu, Jun Wang, Xueke Liu, Mei Lu, Zhenyong Zhang, Lele Li

**Affiliations:** 1 Department of Clinical Laboratory, The Fourth Affiliated Hospital of School of Medicine, and International School of Medicine, International Institutes of Medicine, Zhejiang University, Yiwu, China; 2 Department of Radiology, The Fourth Affiliated Hospital of School of Medicine, and International School of Medicine, International Institutes of Medicine, Zhejiang University, Yiwu, China

**Keywords:** antigen design, infectious diseases, lipid nanoparticles, mRNA vaccines, viral immunity

## Abstract

As a next-generation vaccine technology, mRNA vaccines mark a transformative advancement in vaccinology research. Their key strengths lie in rapid development cycles, high-efficiency manufacturing processes, robust immunogenic properties, and adaptable design frameworks. For infectious disease prevention, mRNA-based immunizations targeting SARS-CoV-2, such as the mRNA-1273 vaccine, have shown promising results in clinical evaluations. Validated efficacy metrics and safety profiles have supported their regulatory approval for public use. Additionally, mRNA platforms have shown substantial therapeutic potential against respiratory syncytial virus. However, this technology still faces multiple challenges, including poor stability, limited delivery efficiency, high production costs, unclear mechanisms of adverse reactions. Distinct from existing literature that primarily focuses on COVID-19 or specific delivery technologies, this review provides a comprehensive, integrated framework covering molecular design, delivery systems, and clinical translation across multiple viral pathogens, while also critically analyzing current controversies and future directions. This review aims to provide a comprehensive reference for researchers and clinicians in related fields.

## 1 Introduction

Vaccines stand as one of the most impactful and economically viable medical strategies for managing infectious diseases through prevention and control measures. Over the years, vaccine technology has progressed through three distinct generations. As a representative of the third generation, messenger RNA (mRNA) vaccines were initially conceptualized in 1989 and subsequently validated in a mouse model in 1990 (Wolff et al., 1990). Follow-up studies confirmed that mRNA transcribed *in vitro* could trigger specific immune responses *in vivo* (Hoerr et al., 2000). However, in the following years, the clinical translation of mRNA technology progressed slowly, primarily hindered by mRNA instability, excessive innate immunogenicity, and the absence of efficient delivery systems.

At the beginning of the 21st century, advances in nucleotide chemical modification and the successful development of lipid nanoparticles (LNPs) delivery systems enabled the practical application of mRNA vaccines. In 2005, Katalin Karikó and Drew Weissman made a pivotal discovery that substituting natural uridine with pseudouridine (Ψ) in mRNA significantly reduced its immunogenicity and enhanced translation efficiency (Karikó et al., 2005). This breakthrough was instrumental in the development of potent mRNA vaccines, and this achievement was honored with the 2023 Nobel Prize in Physiology or Medicine. LNPs are capable of effectively encapsulating and safeguarding mRNA, promoting cellular uptake via their physicochemical properties, and facilitating critical endosomal escape, thus ensuring efficient cytoplasmic delivery and translation of mRNA (Hou et al., 2021).

The 2020 COVID-19 outbreak served as a pivotal historical juncture in the evolution of mRNA vaccine technology. Fueled by pressing worldwide requirements, mRNA vaccines built on these foundational technologies, like BNT162b2 (Pfizer/BioNTech) and mRNA-1273 (Moderna), were developed and granted authorization at an unparalleled speed. In clinical trials (NCT04368728, NCT04470427), they demonstrated over 90% protective efficacy (Baden et al., 2021; Thomas et al., 2021). This success not only addressed a pressing global public health crisis but also verified the core advantages of the mRNA platform, including rapid response capability, scalable production, potent immunogenicity, and flexible design.

Given that viral pathogens represent a major category of microorganisms, mRNA vaccines have emerged as a powerful tool in combating infectious diseases caused by viruses. This review focuses on mRNA vaccine technologies against viral pathogens, including Severe Acute Respiratory Syndrome Coronavirus 2 (SARS-CoV-2), respiratory syncytial virus (RSV), influenza virus, human immunodeficiency virus (HIV), and emerging viruses such as Zika and Ebola. Emerging next-generation RNA platforms are expanding the mRNA vaccine landscape. Circular RNA (circRNA) vaccines, owing to their exonuclease-resistant closed-loop structure, enable prolonged protein expression. Self-amplifying RNA (saRNA) and trans-amplifying RNA (taRNA) vaccines replicate within host cells to achieve high antigen yields at very low doses. Furthermore, artificial intelligence-driven sequence design and structure prediction are increasingly integrated into antigen optimization and UTR engineering. Rather than merely cataloging recent findings, this review synthesizes advances across molecular design, delivery, and immunology to provide an integrated framework for understanding mRNA vaccine development against viral pathogens.

## 2 Principle of mRNA vaccine technology

The core mechanism underlying mRNA vaccines involves the delivery of mRNA molecules that encode target antigens into the cells of the host. These cells then utilize their own translational machinery to synthesize the antigenic proteins. This mechanism triggers targeted humoral and cellular immune reactions, producing defensive memory B lymphocytes and T lymphocytes. By simulating the cellular production of viral proteins that occurs during natural infection, mRNA vaccines are able to elicit a more thorough and well-balanced immune reaction (Chaudhary et al., 2021; Pardi et al., 2018).

### 2.1 Molecular structure and optimization of mRNA

**FIGURE 1:**
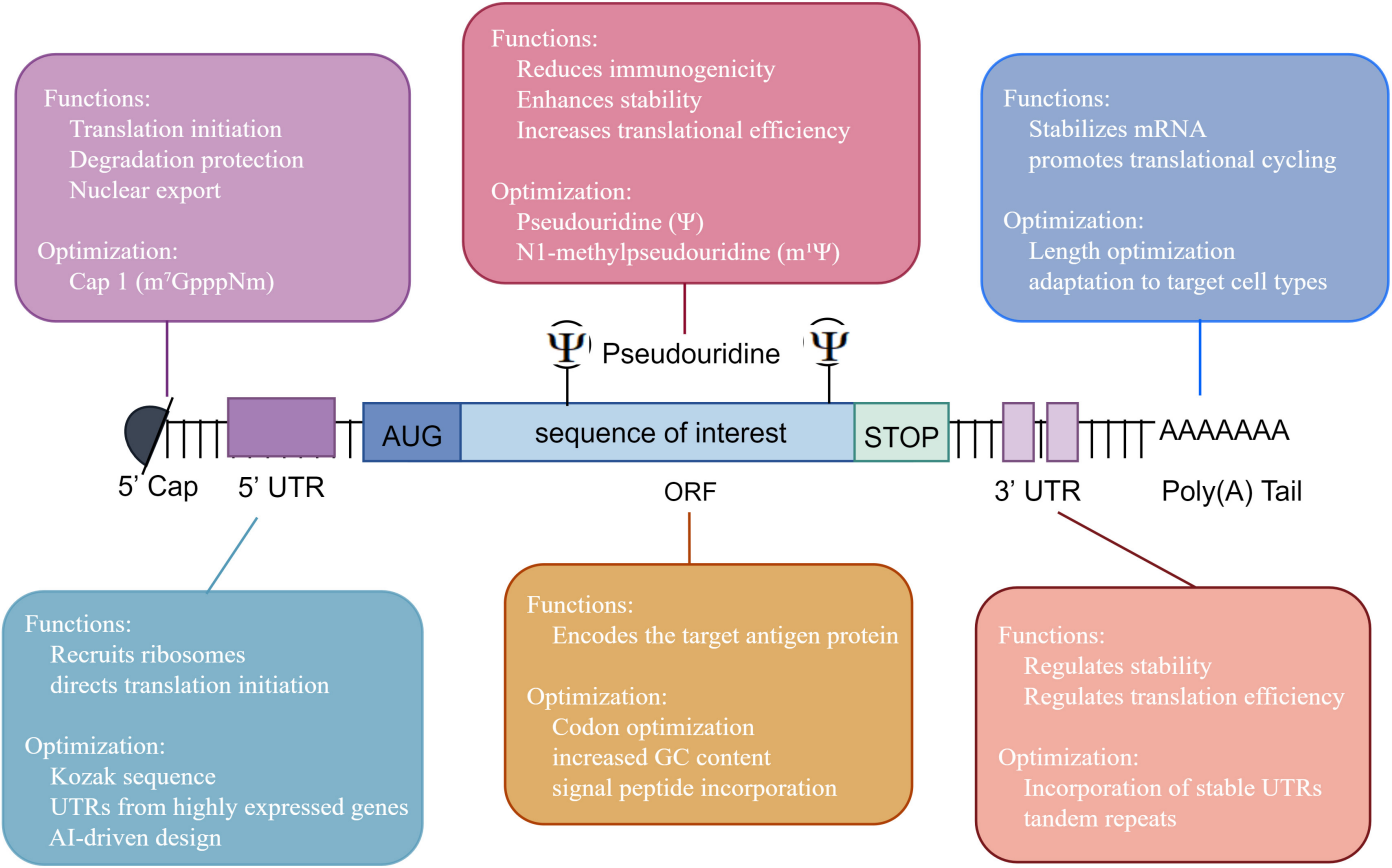
Molecular structure and optimization of mRNA vaccines. *In vitro* optimization of mRNA molecules, including 5′ capping, UTR selection, synonymous codon replacement, synthesis of defined-length poly(A) tails, and uridine substitution with pseudouridine, improves mRNA stability and protein translation efficiency while reducing innate immunogenicity.

The classical structure of a mature mRNA molecule, from the 5′ end to the 3′ end, comprises: the 5′ cap structure (5′ cap), the 5′ untranslated region (5′ UTR), the open reading frame (ORF), the 3′ untranslated region (3′ UTR), and the poly(A) tail. Each component plays a pivotal regulatory role in mRNA stability, translation efficiency, and immunogenicity. Optimizing these elements constitutes a core strategy for improving the efficacy of mRNA vaccines (Figure 1).

#### 2.1.1 5 ′ cap

5′ cap is m^7^GpppN. Its essential chemical feature is 7-methyl guanosine (m^7^G) linked in a reverse orientation via a 5′–5′ triphosphate bridge. Based on the degree of chemical modification, it is primarily categorized into Cap 0 (m^7^GpppN), Cap 1 (m^7^GpppNm), and Cap 2 (m^7^GpppNmNm). Although the Cap 0 structure can mask the 5′-terminal triphosphate, the 2′-OH of its first nucleotide is unmethylated, which may still be recognized as a “non-self” component by specific pattern recognition receptors. The Cap 1 structure achieves more complete “self-camouflage” by methylating the 2′-OH of the first nucleotide. Currently approved mRNA vaccines (BNT162b2 and mRNA-1273) incorporate a Cap 1 analog to ensure effective translation and persistence *in vivo*. The 5′ cap forms a specific binding interaction with eukaryotic translation initiation factor 4E (eIF4E), thereby triggering the process of protein synthesis. It protects mRNA from 5′rom exonuclease digestion, prolongs its half-life (Dolgin, 2021), and is involved in mRNA splicing and nucleoplasmic transport. The 5′ cap can be optimized through enzymatic capping after mRNA transcription or capping during mRNA transcription via co-transcription.

#### 2.1.2 5 ′ UTR and 3′ UTR

5′ UTR and 3′ UTR flank the open reading frame and do not encode proteins. However, they precisely modulate mRNA stability, translation efficiency, and subcellular localization (Orlandini Von Niessen et al., 2019). Located upstream of the start codon, the 5′ UTR is responsible for recruiting ribosomes and guiding the scanning process to the correct translation initiation site (Pardi et al., 2020). It also modulates the translation efficiency of its downstream ORF sequence (Wisse et al., 2017). The 5′ UTR has been optimized through strategies such as introducing the potent Kozak sequence (Kozak, 1987), selecting 5′ UTRs from endogenously highly expressed genes (e.g., the human α-globin gene), and employing artificial intelligence models. For example, the Optimus 5-Prime model developed by Sample et al. (2019) can predict UTR translation efficiency. The secondary structure of the 5′ UTR near the Kozak sequence and start codon critically influences translation initiation. Stable hairpins in the region immediately upstream of and surrounding the start codon can impede ribosome scanning and reduce protein yield. Therefore, minimizing local secondary structures in this region is an important consideration in UTR optimization. 3′ UTRs frequently harbor cis-acting elements that modulate mRNA stability and translational processes, such as AU-rich elements (AREs). The 3′ UTR can be stabilized and optimized by choosing the 3′ UTRs from natural genes (e.g., human albumin or alpha/beta globin genes) (Fang et al., 2022) or by adding 3′ UTR sequences in tandem-repeat form.

#### 2.1.3 ORF

Open reading frame is the core sequence encoding the target antigen, and its design directly influences the expression level and accuracy of the protein. Several sequence-level pitfalls must be avoided during ORF design. CpG motifs require balancing: depletion reduces innate activation, but retention of specific CpGs enhances adjuvanticity. Cryptic splice sites must be eliminated by synonymous mutations to prevent aberrant splicing. Premature polyadenylation signals (e.g., AAUAAA) must be removed to avoid early transcription termination. Without altering the amino acid sequence, replacing rare codons with synonymous codons preferred by host cells can enhance tRNA recognition efficiency, thereby improving the translation rate and protein yield (Mauro and Chappell, 2014). Studies indicate that increasing the GC content of ORFs (typically to 60%–70%) can improve the thermodynamic stability of mRNA molecules and reduce AU-rich regions prone to forming unfavorable secondary structures. This contributes to higher *in vitro* transcription yield and enhanced *in vivo* stability (Brüssow, 2022). By incorporating an appropriate signal peptide sequence into the ORF, the synthesized antigen protein can be directed to a specific cellular compartment (e.g., secreted extracellularly or anchored to the cell membrane), thereby influencing the type of immune response elicited. However, not all proteins require extremely high translation efficiency, as excessive speed may lead to protein misfolding. Therefore, optimization strategies must balance improving translation rates with ensuring correct protein folding (Schlake et al., 2012).

#### 2.1.4 The poly(A) tail

The poly(A) tail, comprised of tens to hundreds of adenylate residues, plays a key role in mRNA metabolism. Its main function is to bind the Poly(A) binding protein (PABP). Together with the 5′ cap, this facilitates the formation of a closed-loop mRNA structure, which enhances mRNA stability by protecting against 3′→c′ exonuclease degradation. Furthermore, this configuration promotes ribosomal recycling and enables continuous translation (Xu et al., 2020). The length of the poly(A) tail serves as a crucial parameter influencing mRNA stability and expression levels. However, a longer poly(A) tail is not always advantageous. The optimal tail length depends on the target cell type (Kwon et al., 2018). Research has shown an optimal length window (about 120 bp) within which protein expression increases with tail length. Beyond this threshold, the gains become marginal or may even decline owing to impacts on delivery efficiency (Fang et al., 2022). As a result, the length of the poly(A) tail needs to be systematically optimized for different target cell types and therapeutic purposes.

#### 2.1.5 Chemical modification of nucleotides

Chemical modification of nucleotides is a core technology for reducing the innate immunogenicity of mRNA while enhancing its stability and translation efficiency. The ORF is the primary and most critical site for modification. The most common strategy involves replacing natural uridine or cytidine with chemically modified nucleotides, such as pseudouridine (Ψ), N1-methyl pseudouridine (m^1^Ψ), or 5-methyl cytidine (m5C). These modifications help mRNA evade recognition by pattern-recognition receptors like TLR7/8 and RIG-I, thereby significantly reducing type I interferon production and inflammatory responses (Iavarone et al., 2017; Pardi et al., 2017). This not only significantly improves the safety of mRNA vaccines but also alleviates the inhibitory effect of interferon on translation, ultimately achieving dual optimization of low immunogenicity and high protein expression. Currently, the approved mRNA-1273 vaccine and BNT162b2 vaccine utilize m^1^Ψ modification technology (Rezaei and Nazari, 2022).

### 2.2 Types of mRNA vaccines

Based on molecular structure and mechanism, mRNA vaccines are primarily classified into three types: non-replicating mRNA (nrRNA) vaccines, self-amplifying mRNA (saRNA) vaccines, and trans-amplifying mRNA (taRNA) vaccines (Table 1).

**TABLE 1:** Characteristics of action and applications of three mRNA vaccine types.

Types of vaccines	Characteristics of action	Clinical applications in infectious diseases
nrRNA vaccines	Direct translation, no replication; Encoding only the target antigen; Antigen expression was high but transient.	SARS-CoV-2 (Corbett et al., 2020), RSV (Aliprantis et al., 2021), Influenza virus (Arevalo et al., 2022), HIV (Zhang et al., 2021), Rabies virus (Li et al., 2023), EBV (Oladipo et al., 2024)
saRNA vaccines	Self-replication, amplifying antigen expression; Encoding target antigen and viral replicase; High and persistent antigen expression.	SARS-CoV-2 (Saraf et al., 2024), RSV (Zuo et al., 2025), Influenza virus (Vogel et al., 2018), HIV (Aldon et al., 2021), Zika virus (Luisi et al., 2020).
taRNA vaccines	Replicase acts in trans to amplify antigen expression; Encoding target antigen and replicase, respectively. The antigen expression was high and persistent.	SARS-CoV-2 (Gontu et al., 2025), Chikungunya virus (Schmidt et al., 2022), Influenza virus (Beissert et al., 2020)

SARS-CoV-2, Severe Acute Respiratory Syndrome Coronavirus 2; RSV, respiratory syncytial virus; HIV, human immunodeficiency virus; EBV, Epstein-Barr Virus.

#### 2.2.1 nrRNA

Non-replicating mRNA vaccines are the most mature and widely used type. They enable peak antigen production within hours to days after administration, making them suitable for scenarios that require the rapid induction of strong immune responses. Key advantages include encoding only the target antigen, having a relatively small size (typically 2–5 kb), a simple structure, mature production processes, and broad compatibility with most antigens or therapeutic proteins. As nrRNA vaccines do not encode any extra functional proteins beyond the antigen, the risk of triggering unnecessary immune responses remains low. Because nrRNA molecules cannot replicate themselves, the yield of the target protein consequently depends on both the cellular uptake of nrRNA and the rate at which it is translated (Beissert et al., 2020). Additionally, the duration of antigen expression is relatively brief, typically lasting from days to 2 weeks, which can present challenges in achieving effective immunity. It often requires a relatively high initial dose and may necessitate booster vaccination to maintain long - term protection (Maruggi et al., 2019).

#### 2.2.2 saRNA

Self-amplifying RNA vaccines originate from the genomic architecture of positive-sense RNA viruses, with alphaviruses serving as a representative example. Their core feature is that, in addition to the ORF encoding the target antigen, they contain genes encoding the non-structural proteins of the viral RNA replicase complex. saRNA vaccines can replicate themselves using their own encoded replicase. The replicase complex recognizes conserved sequence elements (CSEs) on the saRNA, synthesizes a negative-sense intermediate, and transcribes multiple copies of subgenomic mRNA encoding the target antigen. This amplification enables high antigen expression from a very low initial dose. The vaccination of a large population within a short period using a very low initial dose (Rappaport et al., 2022; Cohen, 2022), achieving long-term, high-level antigen expression and potentially providing full protection with a single dose. Research findings indicate that saRNA vaccines directed against influenza viruses are capable of eliciting equivalent immune reactions at a dosage merely 1/64th of that needed for nrRNA vaccines (Vogel et al., 2018). However, the larger size and greater sequence complexity of saRNA vaccines demand higher packaging and transfection efficiency from the delivery system. The double-stranded RNA intermediates produced during the replication process can induce robust innate immune reactions and undesirable inflammatory responses, which may potentially suppress translational activity. Thus, optimizing saRNA vaccines sequences to balance efficacy and safety is a key research focus.

#### 2.2.3 taRNA

Trans-amplifying RNA vaccines are a modular derivative of saRNA vaccines, designed to tackle the issues of their large size and non-target immunity potentially elicited by replicases. Its core design is to insert the sequences encoding the replicase and the antigen protein into two separate expression templates (Rosa et al., 2021), thus modularizing the replication function. This makes the production of taRNA vaccines more flexible, since shorter mRNAs are easier to synthesize and purify. Different antigens can be flexibly combined without the need to redesign the replicase sequence for each. Antigen expression levels can be finely tuned by regulating the replicase dose or activity. Preclinical studies show that taRNA vaccines against influenza effectively induce protective antibodies in mice (Beissert et al., 2020). Consequently, the major challenge is to ensure that two separate templates are efficiently and synergistically delivered to the same cell and optimizing their ratio for optimal amplification.

### 2.3 Delivery systems for mRNA vaccines

Messenger RNA molecules are negatively charged and large (typically 1–15 kb), which prevents them from autonomously crossing the negatively charged lipid bilayer of the cell membrane. They are also susceptible to rapid degradation by ubiquitous ribonucleases (RNases; Schlake et al., 2012). Even when internalized via endocytosis, naked mRNA frequently becomes entrapped in endosomes, thereby failing to reach the cytoplasm for translation (Müntjes et al., 2021). Therefore, developing safe and efficient delivery systems that protect mRNA, promote cellular uptake, and facilitate endosomal escape is essential for stimulating robust immunity. A variety of delivery systems have been engineered, such as lipid-based nanoparticles (LNPs), polymeric nanoparticles, peptide carriers, cationic nanoemulsions (CNEs), biomimetic carriers, and viral vectors.

#### 2.3.1 LNPs

Lipid nanoparticles are acknowledged as the most well-developed and clinically validated mRNA delivery system thus far. Their success stems from rational design and optimization, particularly of ionizable lipids (Huang et al., 2022). A typical messenger RNA-loaded lipid nanoparticle (mRNA-LNP) comprises ionizable/cationic lipids, phospholipids, cholesterol, and polyethylene glycol (PEG), encapsulating mRNA in its core (Taina-González and de la Fuente, 2022; Chaudhary et al., 2021). These components work together to encapsulate, stabilize, deliver the mRNA, and modulate its pharmacokinetics.

Ionizable/cationic lipids constitute the functional core of LNPs, accounting for about 35%–50%. Ionizable lipids are favored over permanently cationic lipids because of their pH-responsive characteristics. They remain neutral at physiological pH (7.4), which minimizes nonspecific interactions and toxicity. In acidic endosomes pH (4.5–6.5), their amino groups become protonated, acquiring a positive charge. This facilitates electrostatic binding to mRNA and interaction with the endosomal membrane, thereby promoting endosomal escape and subsequent mRNA release (Ramishetti et al., 2020). In contrast, permanently cationic lipids retain a positive charge, thereby increasing the risks of membrane destabilization and cytotoxicity (Cui et al., 2018). Phospholipids, as structural lipids, account for about 10%. They are usually neutral, which mainly constitute and stabilize the lipid bilayer structure of LNP (Samaridou et al., 2020; Vishweshwaraiah and Dokholyan, 2022), providing structural support for the entire system. They affect the size, homogeneity, rigidity, and phase transition temperature of nanoparticles (Cheng and Lee, 2016). Cholesterol is the most abundant lipid in LNP formulations, accounting for about 20%–50%. As a neutral lipid, cholesterol is closely inserted into the lipid bilayer. This notably improves the stability and structural integrity of LNP through modulation of membrane fluidity and microdomain structure (Samaridou et al., 2020; Vishweshwaraiah and Dokholyan, 2022). Additionally, it participates in mediating the fusion process of LNP and the endosomal/cell membrane, thereby assisting the cytosolic release of mRNA (Patel et al., 2020). PEG is located on the surface of LNPs, accounting for about 1.5%. Its hydrophilic PEG chains extend on the surface of LNPs, effectively preventing particle aggregation through the steric effect, improving colloidal and storage stability (Kalyanram et al., 2022; Eygeris et al., 2022), and prolonging the *in-vivo* circulation time. The length of the PEG chains and the lipid anchoring sites influence the retention time of PEG on the particle surface, thereby regulating the uptake efficiency of LNPs by immune cells. Additionally, PEG-lipids can attach specific ligands to LNPs to facilitate targeted drug delivery.

Lipid nanoparticles also modulate innate immune activation and biodistribution. Ionizable lipids can activate the STING pathway or induce pro-inflammatory cytokines, acting as an intrinsic adjuvant. PEG density and anchor length affect protein corona formation, phagocyte uptake, and circulation half-life. Particle size determines fate: LNPs < 100 nm drain to lymph nodes, engaging B cells and dendritic cells, while larger particles (>200 nm) remain at the injection site. Tuning these parameters enables targeted biodistribution and desired immune polarization.

The mRNA vaccines approved by The U.S. Food and Drug Administration (FDA), including BNT162b2 and mRNA-1273, employ LNP delivery technology, which has proven reliable and effective in inducing strong humoral and cellular immunity. LNPs provide numerous benefits for mRNA delivery, including a relatively straightforward and scalable preparation process, efficient encapsulation and protection of mRNA, excellent *in vitro* and *in vivo* transfection efficiency, as well as modular compatibility with mRNAs of various sequences and lengths (Chaudhary et al., 2021). However, LNPs also face challenges and limitations. They are susceptible to allergic reactions, oxidative degradation, and there are difficulties in reproducing the preparation process (Trougakos et al., 2022; Szebeni et al., 2022). A major concern is that components of LNPs might elevate the likelihood of hypersensitivity reactions. Current evidence indicates that the potent immunogenicity of cationic or ionizable lipids serves as a primary factor in allergic reactions (Moghimi, 2021). Moreover, a growing body of studies reveals that PEG immunogenicity might also elicit allergic responses upon repeated administration (Kuehn, 2021). Allergic responses noted after administration of mRNA-1273 and BNT162b2 vaccines are probably caused by anti-PEG antibodies generated after the first immunization (Thomas et al., 2021; Baden et al., 2021). However, whether anti-PEG antibodies or complement activation is the primary cause remains controversial.

To tackle these challenges, researchers have explored strategies like designing ionizable lipids with biodegradable linkers or employing alternative lipids (e.g., polysarcosine) to stabilize LNPs and mitigate immunogenicity and toxicity. Certain researchers have additionally utilized the selective organ targeting (SORT) approach to accurately transport mRNA to particular cells, tissues, organs, or tumors through the conjugation of molecules like sugars, nucleic acids, peptides, antibody fragments, or complete antibodies on the LNP surface (Lu et al., 2024).

#### 2.3.2 Polymeric nanoparticles

Polymer nanoparticles can be classified into cationic and anionic structures. Among them, cationic polymers are more suitable for mRNA delivery, as they can spontaneously combine with negatively charged mRNA via electrostatic interactions to form stable polyplexes (Kowalski et al., 2019). The most typical cationic polymer is polyethylenimine (PEI), which can combine with phosphate groups of nucleic acids to generate polycomplex nanoparticles, thus improving transfection efficiency and providing protection for mRNA (Howard et al., 2006). However, as the molecular weight increases, the toxicity and transfection efficiency of PEI also rise. To resolve this problem, Blakney et al. (2020) engineered a bioerodible positively charged polymeric material, pABOL, a poly(amine-co-β-amino ester) polymer, which maintains high transfection efficiency at higher molecular weights. Although the cytotoxicity did not significantly decrease with increasing molecular weight, the polymer was designed to degrade into low-molecular-weight fragments after completing its delivery function (Blakney et al., 2020).

#### 2.3.3 Peptide carriers

Peptide carriers utilize short peptides with positive charges or membrane-penetration capabilities (such as protamine and its derivatives, cell-penetrating peptides (CPPs) to bind with mRNA and form complexes. Protamine can bind tightly to mRNA through strong electrostatic interactions and effectively protect it from nuclease degradation (Hoerr et al., 2000). However, such tight binding can impede the recognition and translation of mRNA by ribosomes, thereby restricting the expression of antigen proteins. To address this limitation, the innovative RNActive^®^ technology platform has been developed. This platform physically mixes protamine with naked antigen-encoding mRNA, which is accountable for the efficient expression of the target antigen. Meanwhile, the protamine-mRNA complex acts as an immune system stimulator, triggering adaptive immune responses via TLR7-mediated signaling (Kallen and Theß, 2014). CPPs are short peptides capable of directly crossing the cell membrane or facilitating endocytosis. CPPs have unique amino acid sequences, allowing them to mediate intracellular delivery more efficiently. To further enhance the delivery efficiency and safety of peptide carriers, Xu et al. (2023) developed a pegylated cationic peptide, which significantly improved the delivery efficiency of mRNA in a mouse model and exhibited better safety.

#### 2.3.4 CNEs

Cationic nanoemulsions are delivery systems in which cationic lipids are incorporated into an oil-in-water nanoemulsion adjuvant. The positive surface charge of CNEs, which originates from the cationic lipids, facilitates electrostatic adhesion to cell membranes with negative charge, consequently boosting cellular internalization and improving delivery performance. Simultaneously, the hydrophobic oil-phase core is capable of encapsulating mRNA molecules, protecting them from nuclease degradation during delivery and thus improving their stability (Shi et al., 2022). MF59, an FDA-approved oil-in-water emulsion adjuvant, has been demonstrated in preclinical studies to successfully deliver mRNA vaccines by integrating the CNE platform with the cationic lipid 1,2-dioleoyl-3-trimethylammonium-propane (DOTAP). This approach has also been shown to induce specific immune human immunodeficiency virus (HIV), human cytomegalovirus (hCMV), and responses against respiratory syncytial virus (RSV) in animal models (Ho et al., 2021). CNEs also offer improved thermostability (2 °C–8 °C storage); other emerging thermostable approaches include lyophilized LNPs, dry powder formulations, and ACM-based platforms.

#### 2.3.5 Biomimetic vectors

Biomimetic vectors are an emerging class of delivery systems, and exosomes are among the most representative biomimetic carriers. The core of their design is to imitate or utilize natural biological structures, endogenous substances, or physiological processes to achieve efficient and intelligent delivery (Yan et al., 2022). Such carriers optimize self-disguise, providing high biocompatibility, low immunogenicity, and potentially inheriting natural targeting and barrier-crossing capabilities. However, they exhibit low delivery efficiency, face difficulties in standardization and large-scale production, and remain in the early research and development stage.

#### 2.3.6 Viral vectors

Viral vectors are created by inserting the target-antigen gene into the genome of an attenuated or replication-defective modified virus. They utilize the virus’s efficient infection mechanism to enter target cells and express the antigen (Wadhwa et al., 2020). At present, viral vectors employed for mRNA delivery mainly consist of viral replicon particles (VRPs) and virus-like particles (VLPs). The most commonly utilized VRPs originate from positive-strand RNA viruses, including flaviviruses, picornaviruses, and alphaviruses. The replicative features of these viruses enable them to act as adjuvants and stimulate potent antibody responses when used as vectors (Chang et al., 2022). VLPs are self-assembled capsids formed by viral structural proteins without containing viral genetic material, thus rendering them non-infectious. In recent years, innovative strategies for loading mRNA into VLPs have emerged. For instance, engineered VLPs can be created by fusing the nucleocapsid protein with the bacteriophage MS2 coat protein, enabling specific recognition and packaging of mRNA bearing an MS2 hairpin structure (Unti and Jaffrey, 2024). This approach harnesses the efficient infectivity of viruses for mRNA delivery while avoiding the potential risks associated with viral genomes. Further research focuses on developing enveloped virus-like particles (eVLPs), which contain structural proteins embedded within a viral envelope, thus combining the characteristics of mRNA vaccines and protein-nanoparticle vaccines (Hoffmann et al., 2023).

### 2.4 Mechanisms of immune activation

**FIGURE 2:**
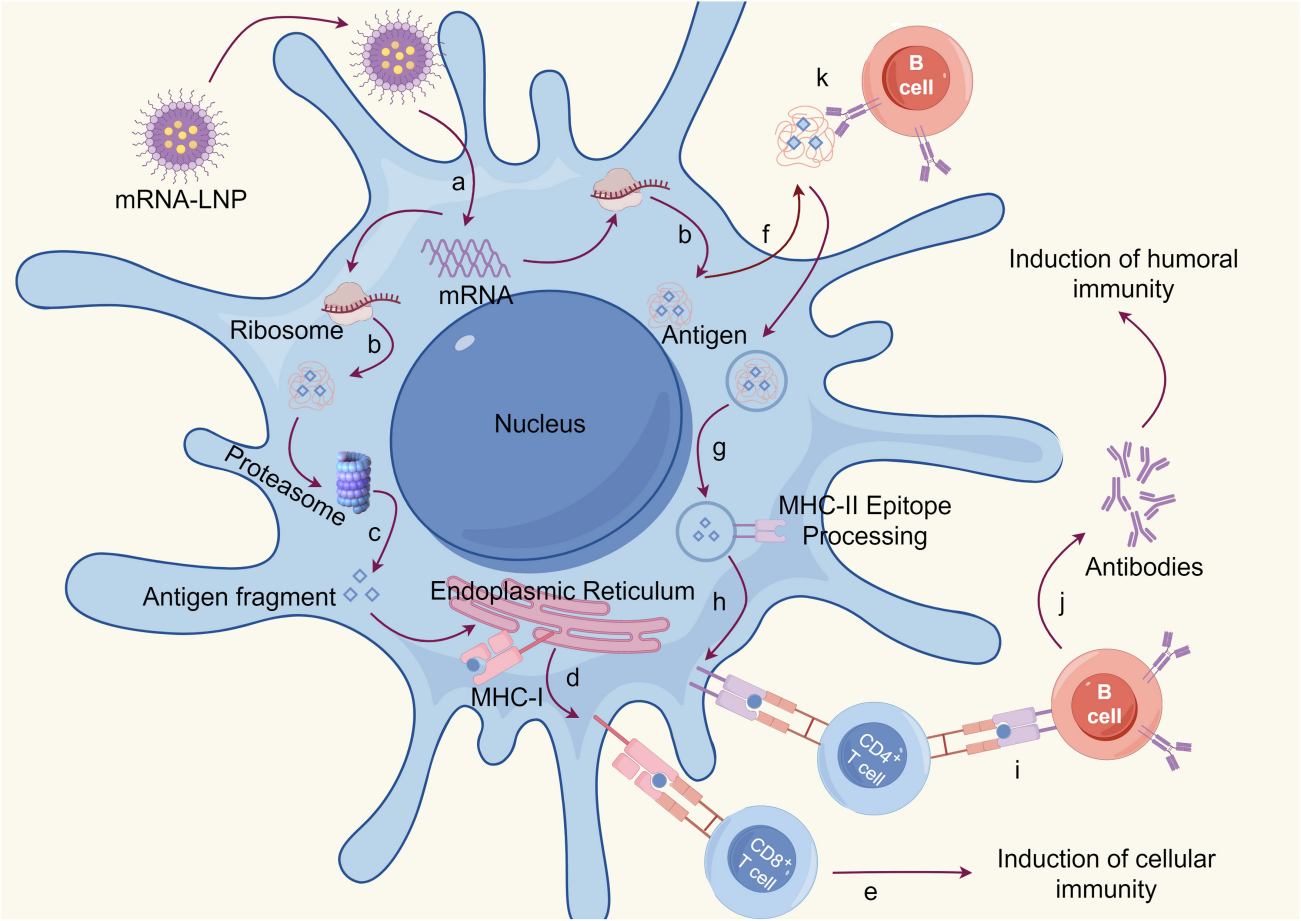
Immune activation mechanisms of mRNA-LNP vaccines. (a) LNP-mRNA endocytosis and cytoplasmic release. (b) mRNA translation into antigen protein. (c) Partial antigen degradation by the proteasome. (d) Peptide loading onto MHC class I molecules and surface presentation. (e) CD8^+^ T cell recognition and activation into CTLs, inducing cellular immunity. (f) Antigen release into the extracellular space. (g) Antigen capture by APCs and lysosomal degradation. (h) Peptide loading onto MHC class II molecules and surface presentation. (i) CD4^+^ Th cell activation and assistance for B cell differentiation into plasma cells and memory B cells. (j) Antibody secretion by plasma cells, eliciting humoral immunity. (k) Direct antigen recognition by B cells.

Messenger RNA vaccine immune activation is a highly coordinated, multi-step process involving both innate and adaptive immunity. Taking an intramuscularly administered LNP-mRNA vaccine as an example (Figure 2).

After vaccine injection, LNP-mRNA complexes are mainly retained at the injection site (e.g., the deltoid muscle) and taken up by local antigen-presenting cells (APCs) (e.g., dendritic cells) or by surrounding muscle cells. Within the cell, the LNP triggers membrane fusion or, upon disruption in an acidic endosomal environment, releases the mRNA into the cytoplasm. Subsequently, the mRNA is translated by ribosomes to produce the target antigen protein. This process is crucial as it represents the fundamental difference between mRNA vaccines and traditional vaccines, since it mimics the protein synthesis pathway that occurs during natural virus infection.

In general, antigen expression and immune response magnitude increase with mRNA dose up to an optimal range, beyond which further dose escalation may not improve immunogenicity and could even trigger suppressive interferon responses. The kinetics of antigen expression also influence the quality of immunity: a rapid, high peak favors strong effector responses, while sustained low-level expression may promote tolerance. These quantitative relationships guide dose selection and regimen design for mRNA vaccines.

A portion of the antigen proteins is degraded by the proteasome, leading to the generation of short peptides. These peptides are translocated into the endoplasmic reticulum via the transporter associated with antigen processing (TAP) and then bound to MHC class I molecules for cell surface presentation. CD8^+^ T cells identify the peptide–MHC I complex through the T-cell receptor and, upon receiving costimulatory signals, get activated and develop into cytotoxic T lymphocytes (CTLs), thus inducing cellular immunity. CTLs can directly eliminate cells expressing the same antigen, which is critical for clearing intracellular pathogens.

Other antigen proteins can be released into the extracellular space via cell death, injury, or active secretion. These extracellular proteins are captured by APCs, degraded into peptides within lysosomes, and presented on the cell surface in association with MHC class II molecules. This pathway activates CD4^+^ T helper (Th) cells. Activated Th cells (especially Th1 and follicular helper T cells) secrete cytokines that provide essential signals for the activation, proliferation, and differentiation of CTLs and B cells (Cui et al., 2021). B lymphocytes directly identify antigen proteins via their surface B-cell receptors (BCRs). With support from activated Th cells, B cells undergo clonal expansion, antibody isotype switching, and affinity maturation, eventually developing into both plasma cells and memory B cells. Plasma cells generate and secrete high-affinity neutralizing antibodies that play a key role in mediating humoral immune responses. Additionally, exogenous mRNA taken up by APCs can be detected by intracellular pattern-recognition receptors (e.g., TLR3, TLR7/8, and RIG-I), leading to type I interferon production and contributing to the self-adjuvant effect of mRNA vaccines (Islam et al., 2021). Mechanistically, excessive type I interferon activates PKR, which phosphorylates eIF2α to block translation initiation, and induces OAS, which activates RNase L leading to mRNA degradation. This interferon threshold must be carefully balanced to avoid translational suppression. However, excessive interferon (IFN) can impair APC function and suppress antigen-protein translation (Linares-Fernández et al., 2020).

The immune responses elicited by mRNA vaccines closely mimic those induced by natural viral infection, yet with key differences. While natural infection exposes the host to the entire viral proteome and may trigger immunosuppressive mechanisms, mRNA vaccines selectively deliver optimized antigen sequences, focusing the immune response on protective epitopes. The induction of virus-specific CD8^+^ T cells through MHC class I cross-presentation is particularly critical for clearing intracellular viral reservoirs. Furthermore, the balance between Th1 and Th2 responses, influenced by mRNA modifications and delivery systems, determines the quality of antiviral immunity. Excessive Th2 skewing has been associated with vaccine-associated enhanced respiratory disease (VAERD) in some preclinical models, highlighting the importance of immune polarization in vaccine safety. This virus-specific T cell immunity, particularly CD8^+^ CTL responses, is essential for eliminating infected cells and establishing long-term immunological memory against viral rechallenge. These virus-specific immune mechanisms are particularly critical for generating long-lived plasma cells and memory B cells that can respond rapidly upon viral rechallenge. The quality of the antibody response, including affinity maturation and isotype switching, directly correlates with protective efficacy against viral pathogens. Thus, compared to natural infection, mRNA vaccination provides focused antigen selection, no viral immune evasion, and a safer, more controllable immune profile.

## 3 Clinical applications of mRNA vaccines in infectious diseases

The swift and successful rollout of mRNA-based vaccines targeting COVID-19 has not only showcased their substantial promise in addressing the global health crisis but also underscored the extensive potential of this technological framework. Looking ahead, the application range of mRNA technology is quickly extending beyond the domain of conventional preventive vaccines, thus driving the advancement of therapeutic vaccines and other areas (Table 2).

**TABLE 2:** Clinical trials of mRNA vaccines targeting viral infectious diseases (clinicaltrials.gov, accessed Jan 15, 2026).

Disease	NCT number	mRNA vaccine	Sponsor	Phases	Status
SARS-CoV-2	NCT05057182	BNT162b2	The University of Hong Kong	PHASE4	Unknown status
SARS-CoV-2	NCT04978038	BNT162b2	Mark Loeb	PHASE4	Unknown status
SARS-CoV-2	NCT06585241	mRNA-1273	ModernaTX, Inc.	PHASE4	Recruiting terminated
SARS-CoV-2	NCT04760132	BNT162b2	Jens D Lundgren, MD	PHASE4	Completed
SARS-CoV-2	NCT04792567	BNT162/mRNA-1273	Novartis Pharmaceuticals	PHASE4	Completed
SARS-CoV-2	NCT06065176	Novavax COVID-19 vaccine (2023-2024 formula XBB containing)/Pfizer COVID-19 mRNA vaccine (2023-2024 formula XBB containing)	Sarang K. Yoon, DO, MOH	PHASE4	Completed
SARS-CoV-2	NCT06020118	mRNA-1273	Duke University	PHASE4	Completed
SARS-CoV-2	NCT06633835	BNT162b2	Novavax	PHASE4	Active, not recruiting
SARS-CoV-2	NCT05142319	BNT162b2/mRNA-1273	Tan Tock Seng Hospital	PHASE4	Completed
SARS-CoV-2	NCT07287137	BNT162b2	Henry M. Jackson Foundation for the Advancement of Military Medicine	PHASE4	Recruiting
SARS-CoV-2	NCT04952402	mRNA-1273/BNT162b2	National Institute of Allergy and Infectious Diseases (NIAID)	PHASE4	Completed
SARS-CoV-2	NCT05030974	mRNA-1273	University Medical Center Groningen	PHASE4	Completed
SARS-CoV-2	NCT04969250	mRNA-1273/BNT162b2	National Institute of Allergy and Infectious Diseases (NIAID)	PHASE4	Completed
SARS-CoV-2	NCT05212610	BNT162b2	University of Michigan	PHASE4	Completed
SARS-CoV-2	NCT04961229	BNT162b2	Dafna Yahav	PHASE4	Unknown status
SARS-CoV-2	NCT07266558	mRNA-1273/mRNA-1283	ModernaTX, Inc.	PHASE4	Recruiting
SARS-CoV-2	NCT06038617	BNT162b2/mRNA-1273	Duke University	PHASE4	Completed
SARS-CoV-2	NCT05343871	BNT162b2	Albert B. Sabin Vaccine Institute	PHASE4	Completed
SARS-CoV-2	NCT04588480	BNT162b2	BioNTech SE	PHASE4	Completed
SARS-CoV-2	NCT04878211	BNT162b2/mRNA-1273	Novartis Pharmaceuticals	PHASE4	Terminated
RSV	NCT07185399	mRESVIA	Emory University	PHASE4	Recruiting
RSV	NCT06067230	mRNA-1345	ModernaTX, Inc.	PHASE3	Active, not recruiting
RSV	NCT06060457	mRNA-1345	ModernaTX, Inc.	PHASE3	Completed
RSV	NCT05330975	mRNA-1345 / mRNA-1273.214	ModernaTX, Inc.	PHASE3	Completed
RSV	NCT05127434	mRNA-1345	ModernaTX, Inc.	PHASE2|PHASE3	Completed
RSV	NCT07117487	mRNA-1345	ModernaTX, Inc.	PHASE3	Recruiting
HIV	NCT00672191	AGS-004	Argos Therapeutics	PHASE2	Completed
HIV	NCT02413645	iHIVARNA-01	Judit Pich Martinez, Fundacion Clinic per a la Recerca Biomedica	PHASE1	Completed
HIV	NCT05001373	mRNA-1644/mRNA-1644v2-Core	International AIDS Vaccine Initiative	PHASE1	Active, not recruiting
HIV	NCT06557785	CH505M5 N197D mRNA-gp160/CH505 TF mRNA-gp160	National Institute of Allergy and Infectious Diseases (NIAID)	PHASE1	Active, not recruiting
HIV	NCT05217641	BG505 MD39.3 mRNA/BG505 MD39.3 gp151 mRNA/BG505 MD39.3 gp151 CD4KO mRNA	National Institute of Allergy and Infectious Diseases (NIAID)	PHASE1	Active, not recruiting
HIV	NCT06694753	mRNA-1645-eODGT8/mRNA-1645-CoreG28v2/mRNA-1645-N332GT5	International AIDS Vaccine Initiative	PHASE1	Recruiting
SARS-CoV-2/Influenza	NCT06694389	mRNA-1083	ModernaTX, Inc.	PHASE3	Completed
Influenza	NCT05827978	mRNA-1010	ModernaTX, Inc.	PHASE3	Completed
Influenza	NCT05566639	mRNA-1010	ModernaTX, Inc.	PHASE3	Completed
SARS-CoV-2/Influenza	NCT06097273	mRNA-1083	ModernaTX, Inc.	PHASE3	Completed
Zika Virus	NCT03014089	mRNA-1325	ModernaTX, Inc.	PHASE1	Completed
Zika Virus	NCT04917861	mRNA-1893	ModernaTX, Inc.	PHASE2	Completed
Rabies	NCT02241135	CV7201	CureVac	PHASE1	Completed
Rabies	NCT03713086	CV7202	CureVac	PHASE1	Completed

Due to the abundance of SARS-CoV-2 and RSV clinical trials data, only selected key Phase IV SARS-CoV-2 and Phase III/IV RSV clinical trials are included. Data for trials with status “Recruiting” or “Active, not recruiting” are presented for informational purposes only and do not imply proven efficacy or safety. All conclusions regarding vaccine performance in the main text are derived exclusively from completed trials with published peer-reviewed results (status “Completed”). SARS-CoV-2, Severe Acute Respiratory Syndrome Coronavirus 2; RSV, respiratory syncytial virus,; HIV, human immunodeficiency virus.

Messenger RNA vaccines are particularly well-suited for combating RNA viruses. Their life cycle involves cytoplasmic replication, which is mimicked by mRNA translation; they encode surface glycoproteins (fusion proteins) that are excellent targets for neutralizing antibodies; and their high mutation rates benefit from the rapid update capability of mRNA platforms. That mRNA vaccines are also being explored for bacterial and parasitic pathogens (e.g., toxin-based vaccines, malaria), though these face different challenges such as antigen complexity and the need for different immune effector mechanisms.

### 3.1 mRNA vaccines and infectious disease prevention

#### 3.1.1 SARS-CoV-2

Severe Acute Respiratory Syndrome Coronavirus 2 is a single-stranded, positive-sense RNA virus that belongs to the genus *Betacoronavirus* within the family Coronaviridae (Mortaz et al., 2020). The genome encodes several structural and non-structural proteins, including the envelope (E) protein, the membrane (M) protein, the nucleocapsid (N) protein, and the spike (S) protein. SARS-CoV-2 serves as the etiological agent behind the worldwide COVID-19 pandemic (Hu et al., 2021). The spike (S) protein, a viral surface glycoprotein, is crucial for mediating host cell entry and serves as the primary immunogen for vaccine development (Hoffmann et al., 2020). The spike (S) protein of SARS-CoV-2 is a class I viral fusion protein that undergoes substantial conformational changes to mediate membrane fusion. The identification of critical neutralizing epitopes within the S protein’s receptor-binding domain (RBD) has guided mRNA vaccine design. Notably, the prefusion-stabilized S protein (with 2P mutations, K986P and V987P) adopted from MERS-CoV research has been instrumental in achieving high immunogenicity. BNT162b2 (Pfizer/BioNTech) and mRNA-1273 (Moderna) utilize LNPs to deliver mRNA encoding this stabilized S protein. These vaccines advanced from pathogen genome sequencing to Emergency Use Authorization (EUA) in about 1 year. In phase III trials, they demonstrated protective efficacies of 95% and 94.1%, respectively (Thomas et al., 2021; Baden et al., 2021). Subsequent large-scale real-world studies have continued to confirm their effectiveness. For example, a nationwide study in Israel reported that complete vaccination with BNT162b2 provided greater than 90% efficacy against symptomatic infection, hospitalization, and severe disease (Dagan et al., 2021).

Regarding safety, both vaccines demonstrated an overall favorable safety profile. The primary adverse events reported were mild-to-moderate local or systemic responses, including injection-site soreness, fatigue, and headache, which typically resolved spontaneously within a short time. Despite reports of extremely rare myocarditis/pericarditis primarily in young males, the FDA maintains that vaccination maintains a positive benefit-risk profile for this demographic. For individuals aged 12–29 years, the estimated risk is 39–47 cases of myocarditis per million vaccine doses. However, vaccination is estimated to prevent approximately 11,000 COVID-19 cases, 139 intensive care unit admissions, and 6 deaths per million in this group (Gargano et al., 2021). The exact mechanisms underlying these rare events remain debated, and conflicting hypotheses include molecular mimicry between spike protein and cardiac antigens, as well as direct inflammatory effects of lipid nanoparticle components.

A core advantage of mRNA vaccines lies in their capacity to quickly adapt and evolve in reaction to viral mutations. Faced with Variants of Concern (VOCs) such as Alpha, Delta, and Omicron, updated vaccines can be rapidly developed by updating the coding sequence (Zeng et al., 2020). For instance, bivalent booster vaccines, which encode both the ancestral strain and variant S proteins, such as those targeting Omicron BA.1 and the BA.4/BA.5 sublineages, have been rapidly developed and deployed. Studies have confirmed that such booster doses can substantially enhance and expand the neutralizing antibody response, thereby restoring protective efficacy. Besides the S protein, vaccines targeting more conserved viral proteins (e.g., nucleocapsid N) or non-structural proteins (e.g., Nsp3, 3CL protease) are also under investigation. These approaches aim to induce a broader range of T-cell immunity, which could assist in counteracting viral mutations and eliminating infected cells.

#### 3.1.2 RSV

Respiratory syncytial virus is an RNA virus with a negative-sense genome classified within the Pneumonaviridae family (Collins et al., 2013). It is a primary cause of lower respiratory tract infections, especially affecting infants, young children, and the elderly. The RSV virion features a lipid envelope adorned with the fusion (F) protein, the attachment (G) protein, and the small hydrophobic (SH) protein (Battles and McLellan, 2019). The RSV fusion (F) protein exists in two conformational states—prefusion and postfusion. The metastable prefusion conformation exposes highly neutralizing epitopes (site Ø) that are absent in the postfusion form. Structural elucidation of the prefusion F protein enabled the design of stabilized variants (e.g., DS-Cav1) that maintain the prefusion conformation, which were subsequently incorporated into mRNA-1345 (Crank et al., 2019). This represents a paradigm of structure-based vaccinology applied to a viral pathogen. Inside, the viral ribonucleoprotein complex contains constituent proteins including M2-1 (serving as a transcriptional processivity enhancer) and M2-2 (governing the conversion from transcriptional events to genomic replication) (Battles and McLellan, 2019). The F protein, particularly in its metastable prefusion conformation, is a critical target for inducing high-potency neutralizing antibodies. For a long time, the inherent conformational instability of the F protein presented a major obstacle to RSV vaccine development, since it hindered the generation of potent neutralizing antibody responses. Given the breakthrough in stabilizing the atomic structure and conformation of the F protein, it becomes feasible to rationally design antigens targeting the prefusion conformation of the F protein. Moderna spearheaded the development of an RSV mRNA vaccine, with its candidate mRNA-1777 encoding a stabilized prefusion F protein. This vaccine represented the initial RSV mRNA candidate to advance into Phase I clinical trials. mRNA-1777 demonstrated positive safety and immunogenicity characteristics, with no severe adverse events documented and demonstrating overall good tolerability. Neutralizing antibody geometric mean titers peaked between days 29 and 60 post-vaccination before gradually declining (Aliprantis et al., 2021).

Building on this foundation, Moderna developed mRNA-1345 through further sequence engineering and codon optimization. This candidate encodes an F protein with enhanced stability, leading to improved translation efficiency and immunogenicity (Aliprantis et al., 2021). A pivotal Phase III clinical trial (NCT05127434) enrolled over 35,000 individuals aged 60 years or above. Results showed that mRNA-1345 had a vaccine effectiveness rate of 83.7% against RSV-associated lower respiratory tract disease (LRTD) with two or more symptoms, and 82.4% effectiveness against LRTD accompanied by three or more symptoms. The vaccine exhibited good tolerability, with the majority of adverse events presenting as mild to moderate in intensity (Wilson et al., 2023). Based on excellent Phase III data, the mRNA-1345 vaccine (mRESVIA^®^, Moderna) received FDA approval in May 2024, thereby becoming the world’s first authorized mRNA vaccine targeting non-COVID-19 infectious diseases (Lu et al., 2024). The successful stabilization of the RSV F protein in its prefusion conformation exemplifies how atomic-level structural insights can directly inform vaccine antigen design—a paradigm that has since been applied to SARS-CoV-2 and other viral pathogens. Indeed, this prefusion stabilization principle—using proline substitutions or disulfide bonds—has been extended to HIV-1 Env, influenza HA, and Ebola GP to enhance neutralizing antibody responses.

#### 3.1.3 Influenza virus

Influenza virus mRNA vaccines constitute single-stranded negative-sense RNA viruses that are classified under the Orthomyxoviridae family, capable of causing both seasonal epidemics and global pandemics. Currently, widely used inactivated or live attenuated vaccines primarily target the viral surface hemagglutinin (HA) protein. However, because influenza viruses frequently undergo antigenic drift and shift, vaccine components need to be updated annually, which carries a risk of mismatch with circulating strains (Zost et al., 2017). mRNA technology offers transformative advantages for influenza vaccine development. The expedited design and manufacturing capacities of mRNA vaccines enable better matching of seasonal influenza circulating strains, thereby enhancing preparedness for emerging epidemics and pandemics. Furthermore, the platform’s ability to encode multivalent antigens facilitates the development of seasonal multivalent vaccines or combination vaccines that target multiple subtypes.

Preclinical and initial clinical trials have tentatively confirmed its potential. For instance, A phase I clinical trial (NCT03076385) of mRNA-LNP vaccines targeting H10N8 and H7N9 subtypes demonstrated that low doses (10–25 μg) could induce high titers of hemagglutination inhibition antibodies and neutralizing antibodies, with a favorable safety profile (Feldman et al., 2019). In animal experiments, quadrivalent mRNA vaccines encoding neuraminidase (NA), the conserved stem region of HA, matrix protein 2 (M2), and nuclear protein (NP) protected mice against heterologous influenza virus challenge, highlighting the protective benefit of a multi-target design (Freyn et al., 2020). Based on this advantage, researchers are actively exploring a “universal influenza vaccine” strategy using mRNA platforms. Arevalo et al. (2022) developed a nucleoside-modified mRNA-LNP vaccine expressing HA proteins from 20 known influenza subtypes with the objective of delivering extensive defense against diverse influenza virus strains. At a dose of 50 μg (2.5 μg per antigen), the vaccine induced specific antibodies against all 20 HA in mice and ferrets, and protected against challenge with H1 subtype influenza viruses and heterologous influenza viruses in the vaccine (Arevalo et al., 2022). Additionally, combination vaccines represent an active area of research and development. Companies such as Moderna and Pfizer have conducted clinical trials of dual vaccines for influenza and COVID-19, and even triple vaccines targeting influenza, COVID-19, and RSV.

#### 3.1.4 HIV

Human immunodeficiency virus represents a single-stranded RNA viral entity classified within the Retroviridae family and serves as the etiological agent responsible for acquired immunodeficiency syndrome (AIDS). The virus primarily attacks and depletes CD4^+^ T lymphocytes, resulting in a gradual deterioration of the host’s immune function and predisposing individuals to various opportunistic infections and malignancies. A major challenge in HIV vaccine development is its envelope protein (Env), which exhibits high genetic diversity and dense glycosylation shielding, making it difficult to elicit potent neutralizing antibodies. mRNA technology presents a novel approach to addressing this long-standing issue. Initial exploration centered on dendritic cell (DC) delivery strategies. For example, Gandhi et al. (2016) demonstrated that delivering HIV antigen via mRNA-transfected autologous DCs enhanced CD4^+^ T cell proliferative responses to the Gag protein, although it induced only a transient and weak CD8^+^ T cell response. Jacobson’s research group carried out a phase IIb clinical trial (NCT00672191) where dendritic cells (DCs) were utilized to administer an mRNA vaccine that encodes a multiprotein structure (Gag, Rev, Vpr, and Nef). Although no significant antiviral effect was observed, the vaccine effectively boosted HIV-targeted CD8^+^ T lymphocyte reactions, laying a foundation for the advancement of T cell-oriented therapeutic vaccines (Jacobson et al., 2016).

With advances in delivery technology, virus-like particle (VLP) -based mRNA vaccines have shown better efficacy in non-human primate models. For example, an mRNA vaccine expressing both the membrane-anchored envelope glycoprotein of human immunodeficiency virus type 1 (HIV-1 Env) and simian immunodeficiency virus group-specific antigen proteins (SIV Gag) successfully induced broad virus-neutralizing antibodies and CD4^+^ T lymphocyte reactions in the rhesus monkey model. It also reduced the risk of infection by 79% under multiple low-dose mucosal challenge (Zhang et al., 2021). Currently, the research focus has shifted to utilizing mRNA-LNP platforms for delivering rationally designed immunogens. Preclinical studies have demonstrated that mRNA-LNP vaccines encoding chimeric immunogens can effectively elicit multifunctional T cell responses targeting conserved epitopes of HIV-1 in mice. Ferritin nanotype-displayed immunogen delivered by mRNA-LNP can induce antibodies that can neutralize heterologous HIV-1 isolates in transgenic mouse models (Mu et al., 2022).

#### 3.1.5 Other important infectious diseases

The notable versatility and effectiveness of mRNA vaccine platforms further offer broad potential for developing vaccines against other key infectious diseases. For example, CV7202, a rabies mRNA vaccine developed by CureVac based on its RNActive^®^ platform, demonstrated in a phase I clinical trial (NCT03713086) that doses as low as 1 μg elicited neutralizing antibodies meeting WHO criteria, it stimulated CD4+ and CD8+ T cell responses and exhibited a favorable tolerability profile (Aldrich et al., 2021). In non-human primates, the immunogenicity of the optimized version was even superior to that of the commercially available conventional vaccine. mRNA vaccines targeting other high-risk or common pathogens, such as Zika virus, Ebola virus, cytomegalovirus (CMV), Epstein-Barr virus (EBV), etc., are also in active preclinical or early clinical development. Computational tools, including artificial intelligence-based epitope prediction, are increasingly being integrated into antigen design for emerging viral pathogens, potentially accelerating vaccine development against future outbreaks, although current limitations include training data bias, poor generalization to viral RNA structures, and lack of regulatory guidelines.

## 4 Conclusions and future perspectives

### 4.1 Fundamental concepts and current challenges

In the context of advancing mRNA vaccine strategies for viral immunity, several key challenges and future directions emerge from this synthesis. As summarized above, existing mRNA vaccines still encounter challenges, such as poor stability, which requires ultra-low temperature storage and transportation (Yang et al., 2023). This drawback limits their application in regions with limited resources. The delivery efficiency is still not optimal, as a substantial amount of mRNA is retained and degraded within endosomes, which impairs expression and immune activation. This can lead to local or systemic inflammatory reactions, such as hypersensitivity reactions and myocarditis (Jaggers and Wolfson, 2023; Oster et al., 2022). Additionally, the significant disparities between preclinical models and human immune responses heighten the uncertainties in clinical translation (Bahl et al., 2017).

### 4.2 Controversies and research gaps

Despite the success of mRNA vaccines, several controversies remain. The optimal balance between innate immune activation and antigen expression continues to be debated, as excessive type I interferon responses may suppress translation while adjuvant effects are desirable. The mechanisms underlying rare adverse events, such as myocarditis following mRNA vaccination, are still not fully understood, with hypotheses ranging from lipid nanoparticle components to cross-reactive immune responses. Conflicting evidence exists regarding durability and booster requirements: protection against symptomatic SARS-CoV-2 infection has been shown to wane within months, while protection against severe disease remains longer. For influenza and HIV, durability data are still limited, and no universal rule applies across viral pathogens. Furthermore, the need for booster doses varies by pathogen and population, reflecting these discrepancies.

### 4.3 Future directions and potential developments

Despite these challenges, mRNA vaccine technology holds broad prospects for future development. For viral infectious diseases, the rapid emergence of variants of concern (VOCs) poses a continuous challenge to mRNA vaccine efficacy. The platform’s flexibility enables swift sequence updates, as demonstrated by bivalent boosters targeting Omicron sublineages. Future directions include developing ‘pan-virus’ vaccines targeting conserved viral regions (e.g., influenza HA stem, coronavirus RBD conserved motifs) to preempt viral evolution. Additionally, mRNA vaccines against emerging zoonotic viruses (Nipah, Lassa, etc.) could be stockpiled as pre-pandemic countermeasures, leveraging the platform’s rapid response capability.

In conclusion, mRNA vaccines represent a transformative breakthrough in combating viral infectious diseases. This highly adaptable platform, validated during the COVID-19 pandemic, is now being applied to a widening array of viral pathogens. Although challenges remain regarding stability, delivery efficiency, and rare adverse events, ongoing innovations in sequence design, delivery systems, and manufacturing processes are expected to overcome current limitations. In the future, mRNA technology is poised to become a cornerstone of pandemic preparedness and global infectious disease control, offering virologists and microbiologists a powerful tool to combat evolving viral threats.
